# Sleep in the Social World of College Students: Bridging Interpersonal Stress and Fear of Missing Out with Mental Health

**DOI:** 10.3390/bs10020054

**Published:** 2020-02-06

**Authors:** Sue K. Adams, Karla K. Murdock, Meada Daly-Cano, Meredith Rose

**Affiliations:** 1 Department of Human Development & Family Studies/Professor/College of Health Sciences, University of Rhode Island, Kingston, RI 02891, USA; 2 Department of Psychology, Washington and Lee University, Lexington, VA 24450, USA; murdockk@wlu.edu; 3 Department of Psychology/College of Health Sciences, University of Rhode Island, Kingston, RI 02891, USA; meada_dalycano@uri.edu; 4 Department of Human Development & Family Studies/College of Health Sciences, University of Rhode Island, Kingston, RI 02891, USA; mhrose2@childrensnational.org

**Keywords:** sleep, adolescents, FoMO, stress, mental health

## Abstract

Introduction: The college years are characterized by psychosocial and biological phenomena that may impact mental health, such as heightened sensitivity to social stressors and compromises in sleep quantity and quality. The current study uses a biopsychosocial approach to examine the associations among interpersonal stress, Fear of Missing Out (FoMO), insomnia, and mental health. Methods: Survey data were collected from 283 undergraduate students (90% female) with a mean age of 21.4 years. A path analysis was utilized to test a mediational model linking interpersonal stress and FoMO with mental health through a mediator of insomnia. We hypothesized that higher levels of interpersonal stress and FoMO would be associated with higher levels of insomnia symptoms, which would in turn be associated with poorer mental health. Results: As predicted, insomnia partially mediated significant associations of interpersonal stress and FoMO with mental health. The association of interpersonal stress with insomnia and mental health was more robust than the association of FoMO with these variables. Conclusions: The pathway from interpersonal stress and/or FoMO, through insomnia, to compromises in mental health may be modifiable through behavioral interventions focusing on coping skills, sleep hygiene, and even technology-related habit changes. Recommendations to help disrupt this pathway, particularly among college students, are discussed.

## 1. Introduction

Over the past decade, mental health has become an increasing concern on college campuses across the United States. A recent survey of college counseling centers revealed a 30% increase in self-referrals between 2009 and 2015, with 61% of these undergraduates endorsing anxiety and 49% endorsing symptoms of depression [1]. Unfortunately, this increasing demand tends to outweigh available university counseling resources [2]. Mental health directly and indirectly affects other critical aspects of college student development, such as academic performance, social relationships, and physical health [3,4]. Thus, it is critical to examine the psychological, social, and biological phenomena that characterize college students and may help to explain variance in their mental health.

### 1.1. The Role of Sleep in Maintaining Mental Health

Sleep is a critical biological process that is intimately tied to psychosocial processes. Without adequate sleep, college students are prone to diminished social and cognitive functioning, work absenteeism, decreased physical and mental health, and lower overall quality of life [5,6,7]. Multiple factors associated with the college experience may contribute to sleep deprivation [5]. For example, in two studies examining sleep and technology use among undergraduates, higher levels of nighttime cellphone use and compulsive cellphone use were associated with higher levels of sleep problems [8,9]. 

Despite the importance of sleep, researchers have found that college students sleep an average of only 6 h per night [10]. College students are also at increased risk of insomnia (i.e., difficulty initiating or maintaining sleep), early-morning awakenings, non-restorative sleep, and daytime sleepiness [11]. In a study exploring the prevalence and correlates of insomnia, 8.7% of college students endorsed difficulty falling or staying asleep and related daytime impairments at least three times per week for at least six months [12]. 

There is strong support for associations of poor sleep quality and quantity with mental health concerns. In studies of undergraduate students, those who reported poorer sleep quality or reduced sleep quantity also endorsed elevated rates of depression and/or anxiety [10,13], and those who met criteria for insomnia reported increased rates of stress, fatigue, mental health, hypnotic and stimulant use, and decreased quality of life [12]. A recent longitudinal study found that chronic stress, as well as symptoms of depression, were predictive of sleep onset insomnia and hypersomnia among second-year college students [14], another longitudinal study of college students found that stress and sleep problems were bidirectional [15], and multiple studies have demonstrated that mood disorder diagnoses are often preceded by and predicted by poor sleep [16,17]. The association between stress, disrupted sleep and mental health deserves further research attention in college populations. 

### 1.2. Interpersonal Stress

College students experience an array of transient and persistent types of modifiable stressors [18]. Among these, interpersonal stressors, which are negative experiences involving conflicts or difficulties in social relationships, may be particularly salient [19]. Interpersonal stress tends to arise when individuals place high importance on maintaining relationships and avoiding rejection, and it is associated with increased vulnerability for low self-esteem, anxiety, and depression [13]. These associations may also be transactional, such that individuals with depression may generate stress in their relationships, in turn exacerbating relational stress [20]. 

Social stress appears to be a major contributor to the development of anxiety and depression, and the way that college students cope with this type of stress can be a critical factor in their mental health outcomes [3]. Perceptions of social support are integral to interpersonal satisfaction and psychological well-being, so it stands to reason that social disharmony may negatively impact the health and wellness of young adults [21]. 

Interpersonal stressors such as relationship conflict have been directly associated with sleep problems such as later bedtime, longer onset latency, maintaining and reinitiating sleep, shorter duration, and poor sleep quality [22,23,24]. Gordon and colleagues (2017) report that using an experimental design and actigraphy measures, they found that compared to a neutral experience, exposure to an interpersonal stressor led to later bedtime and shorter sleep duration [25]. Among individuals with insomnia, indicators of interpersonal distress have been associated with increased arousal and insomnia severity [26]. Thus, interpersonal stress is likely to influence mental health outcomes not only directly, but also indirectly through compromised sleep. 

### 1.3. Fear of Missing Out in College Students

In recent years the construct of Fear of Missing Out (FoMO), has emerged to describe an individual’s all-consuming sense that others are engaging in rewarding experiences in their absence [27]. Although much of the current literature correlates FoMO with social media use [28,29,30,31], FoMO as a construct can more broadly be applied to any lack of connectedness or closeness with others. Based on self-determination theory, psychological health and self-regulation are contingent on three basic principles—competence, personal initiative, and connectedness with others [32]. Although achieving these basic needs is easily facilitated by using digital social media tools that promote social connectedness and instant knowledge of what others are doing [27], the anxiety and worry about what others may be doing in your absence can also be a pervasive issue independent of social media use. For instance, a number of anxiety disorders, such as social anxiety disorder and separation anxiety disorder, include dimensions of worry related to what others are doing while out of one’s presence [33]. Moreover, both FoMO and anxiety disorders are inversely correlated with a number of similar negative outcomes, including poor mood, decreased sense of life satisfaction, and dysregulated sleep [27,29,30].

### 1.4. The Current Study

The current study used a biopsychosocial framework [34] to examine associations of interpersonal stress and FoMO with college students’ mental health, and to explore if these associations were partially mediated by sleep problems. Path analysis was utilized to test a theoretical model in which higher levels of interpersonal stress and FoMO were expected to be associated with higher levels of insomnia symptomatology, which in turn were expected to correlate with poorer mental health. 

## 2. Methods

### 2.1. Design and Participants

Data were collected from 283 English speaking undergraduate students (first years, sophomores, juniors, and seniors) enrolled at mid-sized northeastern university. The majority of participants were female (90.0%) and white (85.2%). The participant mean age was 21.4 with a range from 18 to 50. The majority of students were sophomores (32.5%) and juniors (32.9%). Participants were required to be at least 18 years of age. In an effort to increase the diversity of the sample, the sampling goal was 250 students across different majors at the university. A link to an anonymous survey, which addressed technology use and habits, stressors, and overall health and well-being, was provided to the instructors of courses in a variety of majors (e.g., psychology, human development and family studies, nursing, kinesiology). The first page of the survey included Informed Consent, and by continuing with the survey participants consented to participate in the research study. Instructors were asked to provide to the students the link through the university’s online class management system. Each survey took between 15–25 min to complete. Some instructors chose to offer extra credit as an incentive to students who participated, but this was not a requirement. The research was approved by and followed the guidelines set forth by the Institutional Review Board at the university. 

### 2.2. Measures

Participants self-reported all information on an online survey administered in Qualtrics. The mean hours of sleep each night and the amount of sleep disturbances over past two-week period were calculated based on participant self-report. 

*Mental Health.* Mental health was measured using the 5-item mental health subscale from the Medical Outcomes Study (MOS) Short-Form General Health Survey [35]. Sample items included “How much of the time, during the past month, have you been a very nervous person?” or “…felt downhearted and blue?” The responses ranged from 1 (“all of the time”), 2 (“most of the time”), 3 (“a good bit of the time”), 4 (“some of the time”), 5 (“a little of the time”) to 6 (“none of the time”). Two items were reverse scored (e.g., “During the past month, how much of the time have you felt calm and peaceful?”), so that high scores on all items, and on the total score, reflect better mental health. Consistent with Stewart et al. (1988), the scores were transformed linearly to 0–100 scales with 0 and 100 assigned to the lowest and highest scores respectively [35]. An internal consistency of 0.96 was reported for the MOS 38-item general health scale [35], which has been used in samples of young adults [36]. The internal consistency of the five mental health items utilized in this sample was good (α = 0.88).

*Insomnia.* Insomnia symptoms were assessed with the Insomnia Severity Index (ISI) [37], a 7-item self-report questionnaire addressing the nature, severity, and impact of insomnia in the past 2 weeks. The ISI evaluated the following aspects of insomnia: severity of sleep onset difficulties, sleep maintenance, and early morning awakening problems; sleep dissatisfaction; interference of sleep; difficulties with daytime functioning; noticeability of sleep problems by others; and distress caused by the sleep difficulties. A sample item was: “Please report how often you have had these experiences during the last two weeks, - Difficulty falling asleep.” Participants responded on a 4-point scale ranging from 0 (“none”), 1 (“mild”), 2 (“moderate”), 3 (“severe”) to 4 (“very severe”). Responses were summed to form a total score, ranging from 0 to 28 with higher scores indicating greater symptoms of insomnia. Internal consistency of the seven insomnia symptoms in this sample was good (α = 0.86). 

*Fear of Missing Out.* Fear of Missing Out (FoMO) was measured using the 10-item Fear of Missing Out scale [27]. A sample item was: “I get anxious when I don’t know what my friends are up to.” Participants responded on a 5-point scale ranging from 1 (“not at all true of me”), 2 (“slightly true”), 3 (“moderately true”), 4 (“very true of me”) to 5 (“extremely true of me”). Responses were summed to form a total score, ranging from 10 to 50, with higher scores indicating greater fear of missing out. This measure has demonstrated internal consistencies from 0.87–0.90 in previous studies [27]. Internal consistency of the 10 items in this sample was excellent (α = 0.92).

*Interpersonal Stress.* A 25-item interpersonal stress inventory was created for this study, building on the foundation of the Social Stress Questionnaire (SSQ) [38]. In addition to the 10 items on the original SSQ, items address typical stressors of a contemporary college lifestyle (e.g., roommates; drinking/partying; technologically-mediated communication). A sample item in this 25-item questionnaire was “Some of the major hassles and stressors for college students come from relationships with other people… Having problems with people you reside with.” For each experience that was endorsed, respondents were asked to rate its stressfulness during the past month on a 4-point scale ranging from 1 (“has not occurred to me”), 2 (“not at all”), 3 (“a little stressful”), 4 (“somewhat stressful”) to 5 (“very stressful”). A principal axis factor analysis was conducted with extraction of factors based on eigenvalues greater than one. Results indicated that all items loaded onto a single factor, and 40% of the variance was accounted for in this solution. The interpersonal stress variable was calculated by summing stressfulness ratings for all items. The total score ranged from 25 to 125, higher scores indicated greater perceived interpersonal stress during the past month. Internal consistency of this measure was excellent (α = 0.94).

### 2.3. Data Analytic Strategies

Descriptive statistics were calculated using SPSS for Mac, version 24 (IBM Corp., Armonk, NY, USA). Path analyses were conducted using EQS 6 (Multivariate Software Inc., Encino, CA, USA). Missing data were corrected with mean imputation for 12 cases missing less than 5% of the data. Seven cases were dropped due to missing more than 5% of data. Path analysis—an extension of multiple regression—estimated the associations among all specified variables in a single model. Three models were compared to test the hypothesis that fear of missing out and interpersonal stress are associated with insomnia symptoms, which in turn are associated with mental health. The first model tested the direct relationship of interpersonal stress and FoMO with the outcome of mental health. The second model tested the relationship of interpersonal stress and FoMO with the outcome of mental health using insomnia as a mediator. The full model included both of the direct paths and the meditation paths. The data analyses proposed to determine the validity of the full model as well as the strength of relationships between each factor.

## 3. Results

### 3.1. Preliminary Analysis

Descriptive statistics on the variables of FoMo, interpersonal stress, insomnia, and mental health suggested that the assumptions of normality, linearity and homogeneity of variance did not appear to be violated. Means (with standard deviations in parentheses), scale ranges, skewness, kurtosis and zero order correlations of study variables are presented in Table 1. The mean hours of sleep per night over the past two-weeks for participants was 7.4 (SD = 1.0) and the range was 3.4–11. The mean number of sleep disturbances per night over the past two weeks was 8.9 (SD = 5.2) with a range from 0–25. 

### 3.2. Path Analysis

Three models were tested, the direct model, mediation model and full model and results indicated that the full model was the best fit of the three tested models. Model fit was evaluated using the χ^2^, comparative fit index (CFI) and the root mean square error of approximation (RMSEA) with a better fit indicated by CFI > 0.90 and RMSEA < 0.05 [39]. Table 2 presented the main indices of evaluating fit across the three alternate models. The *p*-values of the first two models are significant, and the CFI’s are below 0.90. The RMSEA for both models are greater than 0.05 with wide confidence intervals. All indices showed that the first two models are a poor fit of the data. The Full model has an χ^2^ value of 0.001 and a large *p*-value of 0.99 with a CFI of 1 and RMSEA of 0.00, indicating that this model cannot be rejected. 

**Figure 1:**
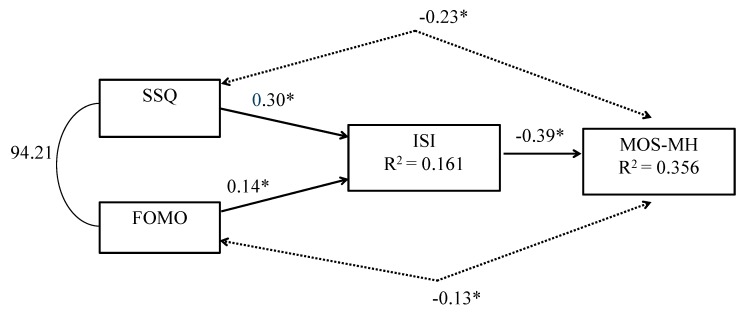
Standardized path coefficients and R^2^ values for full model, * significant at the 0.05 level.

Figure 1 showed the standardized path coefficients and the R^2^ values for the full model. All paths were significant at the 0.05 level. A level of FoMO one standard deviation above the mean was associated with an increase in insomnia 0.14 standard deviations above the mean. A level of interpersonal stress one standard deviation above the mean was associated with insomnia 0.30 standard deviations above the mean, suggesting that interpersonal stress had a stronger relationship with insomnia than FoMO. The R^2^ value of 0.356 in the mental health outcome showed that the relationship of interpersonal stress, FoMo and insomnia accounted for almost half of the shared variance.

## 4. Discussion

The results of the path analysis supported the hypothesized partial mediation model. Higher levels of interpersonal stress and FoMO were associated with higher levels of insomnia, which in turn were associated with poorer mental health. 

Compared to FoMO, interpersonal stress was more robustly associated with insomnia symptoms and mental health. This is perhaps not surprising, as the construct of interpersonal stress encompasses a broad swath of experiences with one’s family, friends, significant other, and people in the larger social world. A majority of young adults must independently navigate new and complex social demands that require the development of sophisticated social skills such as role-taking and conflict management [18,27], and these challenges may generate interpersonal stress across many domains of life. In contrast, FoMO may tend to emerge more readily among a subset of students who have low levels of life satisfaction, self-competence, autonomy, or connection to others, and/or low mood [27], and/or it may be generated in a selective set of interactions or experiences that promote social comparison, such as the use of social media [40]. It is also plausible that since the relationship between a desire for social connectedness and social media use is strongly mediated by FoMO [27], a measure of FoMO related to technology use would have yielded more robust results.

Several psychological, behavioral, social, and/or biological processes may drive the mediational role of insomnia in the association of stress and FoMO with mental health. Importantly, college students who experience FoMO and/or interpersonal stress may bring these experiences to bed with them, remaining in an activated cognitive or emotional state. Negative affect arising from stress may promote or maintain biological processes such as cortisol production and/or autonomic nervous system arousal. These biological states may generate sleep impairment in the short term and/or dysregulation of the HPA axis in the longer term, both of which could facilitate mental health problems related to emotional dysregulation. 

College students experiencing FoMO or interpersonal stress may try to reduce their negative affect or anxiety, or actively cope with their stress, through behavioral means as well. They may consult social media or reach out to sources of social support via cellphone—strategies that may delay sleep onset or lead to co-rumination. If behaviors such as these are crystallized, they may add to the cumulative sleep-related risks often experienced by college students [5,11,12]. They may also produce an independent source of distress and exhaustion that contributes to the individual’s mental health burden.

### 4.1. Study Limitations

This study identifies one pathway through which modern stressors may compromise college students’ mental health. However, the limitations of these findings should be noted. First, all data were self-reported. Objective biomarkers of stress and/or actigraphy-based analysis of sleep would allow a more rigorous and nuanced test of sleep problems that may mediate between stress and mental health. Second, the sample was predominantly female and had limited racial diversity, which limits the generalizability of findings. A meta-analysis of insomnia in the general population suggested that women may have greater problems with insomnia than men, but gender differences in insomnia increased with age [41]. In contrast, a meta-analysis of insomnia in university students found that five studies reported no gender differences in insomnia, one study found greater insomnia in men, while another found greater insomnia in women [42]. Third, mediation models were tested with data collected at one point in time, therefore directionality cannot be determined. Evidence from a longitudinal study suggests that stress and poor sleep quality have a complex bidirectional relationship [15]. Findings of another longitudinal study found that insomnia may be a premorbid risk for incident depression, or self-report of depression [17]. Longitudinal data will be necessary to examine with greater precision the direction/s of effects among stress, FoMO, insomnia symptoms, and mental health. Finally, although multicollinearity between the interpersonal stress and FoMO measures was addressed by standardization in this study, further examination is warranted to clarify whether FoMO is a distinct trait-based construct or rather a state-based stressor. 

### 4.2. Implications and Future Directions

Given the prevalence of mental health concerns on college campuses [5,43], it is imperative to identify mechanisms through which psychological well-being can be promoted, maintained, and/or restored. The current study has identified insomnia as one mechanism through which students’ social stressors may contribute to mental health problems. In keeping with the biopsychosocial model, this suggests that health promotion efforts focusing on sleep habits may help to address mental health outcomes on college campuses. Furthermore, as recently recommended by the American College of Physicians (ACP) [44], college health and counseling centers should include cognitive behavioral therapy in addition to medication management as first choice options for sleep disturbances. Importantly, cognitive behavioral therapy for insomnia has a favorable profile of empirical validation and few side effects [45] and its efficacy in alleviating symptoms of depression and anxiety has been demonstrated [46].

Future research should examine a possible role of college students’ technology use in the associations between stress, sleep, and mental health revealed in the current study. For instance, FoMO and/or interpersonal stress may produce or maintain symptoms of insomnia by encouraging college students to engage with technology in a manner that compromises sleep and/or mental health. Likewise, individuals experiencing interpersonal stress may feel pressure to stay connected to others and/or to the stressful situation via cellphone, which may be problematic for sleep and mental health. Stewing in cellphone-mediated communication may perpetuate stress-related negative effects and physiological arousal, risk the exacerbation of interpersonal stress through misunderstandings and/or co-rumination, and/or prevent a restorative period of peace and objectivity during which effective problem-solving about a stressful situation can occur. Given the pervasiveness of cellphone use among college students [47], it will be important for future research to identify how technology use fits into the associations between interpersonal stress, FoMO, insomnia, and mental health revealed in this study.

## Figures and Tables

**Table 1:** Mean, standard deviations, scale ranges, and Zero Order Correlations of Study Variables.

Variable	1	2	3	4
M (SD)	61.0 (19.0)	22.0 (8.2)	8.0 (5.3)	64.2 (17.5)
Range	25–109	10–45	0–25	12–100
Skewness	0.28	0.58	0.73	−0.36
Kurtosis	−0.46	−0.51	0.06	−0.13
1. Interpersonal stress	—			
2. Fear of Missing Out (FoMO)	0.61 **	—		
3, Insomnia	0.40 **	0.32 **	—	
4. Mental Health	−0.050 **	−0.40 **	−0.52 **	—

** *p* < 0.01 (two-tailed).

**Table 2:** Fit indices for three Path Analysis models, N = 283.

Model	*χ^2^*	*Df*	*p*	*CFI*	*RMSEA*	*90% CI*
Direct Effect	49.64	3	<0.001	0.843	0.24	0.180, 0.294
Mediation	36.65	2	<0.001	0.883	0.24	0.181, 0.342
Full Model	0.001	1	0.99	1.00	0.00	
